# Predominance of *Klebsiella pneumoniae* ST14 carrying CTX-M-15 causing neonatal sepsis in Tanzania

**DOI:** 10.1186/1471-2334-13-466

**Published:** 2013-10-07

**Authors:** Stephen E Mshana, Torsten Hain, Eugen Domann, Eligius F Lyamuya, Trinad Chakraborty, Can Imirzalioglu

**Affiliations:** 1 Catholic University of Health and Allied Sciences-Bugando, Box 1464, Mwanza, Tanzania; 2 Institute of Medical Microbiology, Justus-Liebig University, Schubertstrasse 81, Giessen D-35392, Germany; 3 Muhimbili University of Health and Allied Sciences, Box 65001, Dar es Salaam, Tanzania; 4 German Centre for Infection Research (DZIF), Partner site Giessen-Marburg-Langen, Campus, Giessen, Germany

## Abstract

**Background:**

*Klebsiella pneumoniae* strains expressing ESBLs are a predominant cause of hospital acquired infections. Here we describe the molecular epidemiology of these isolates in a tertiary hospital in Tanzania, as potential pathogens for neonatal infections.

**Methods:**

Between April 2009 and March 2010 all *Klebsiella pneumoniae* isolates with phenotypic expression Extended Spectrum Beta Lactamase (ESBL) were collected and characterized. Identification was done using in house biochemical tests in case of ambiguous results confirmation was done using API 20E. Susceptibility testing was determined using the disc diffusion method followed by specific PCR and sequencing to determine ESBL genes. Phylogenetic analysis, Pulse field gel electrophoresis (PFGE) and Multi-Locus sequence typing (MLST) to PFGE clusters representative isolates were performed to determine clones of the isolates. Conjugation and hybridization were performed to determine the location of blaCTX-M-15 gene.

**Results:**

A total of 92 non- repetitive ESBL producing *K. pneumoniae* representing 50.3% of *Klebsiella pneumoniae* isolates were characterized. These isolates were from blood 61 (66%), wound swab 13 (14%), urine 12 (13%) and pus 6 (7%) were analyzed. Most blood culture strains originated from neonatal unit 39/61(64%) and 22 (36%) of the blood culture isolates were from neonatal ICU. All isolates were resistant to gentamicin and 54% were resistant to ciprofloxacin. Using a similarity index of 80%, the isolates were assigned to thirteen clusters based on PFGE patterns and contained sub-clusters with identical strains indicating clonal outbreaks. Cluster X5, X7 and X8, and X9 were grouped into ST48, ST14 and ST348 respectively. Based on *gyr*A PCR- RFLP phylogenetic analysis all isolates were grouped as KpI. The predominant ESBL allele detected was *bla*_CTX-M-15_ which was found in 76% of isolates, followed by *bla*_TEM-104_ (19%), *bla*_SHV-11_ (3.2%) and *bla*_TEM-176_ (2%). The *bla*_CTX-M-15_ gene was located in multiple conjugative IncF plasmids ranging from 25 kb-485 kb in size.

**Conclusion:**

The high prevalence of *bla*_CTX-M-15_ observed among ESBL producing *K. pneumoniae* in Tanzania, is possibly due to the spread of a common IncFII 145 kb plasmid and of certain clones such as ST14 and ST48. Furthermore the 485 kb plasmid detected is the largest plasmid reported to carry *bla*_CTX-M-15_ todate.

## Background

Gram negative bacteria from clinical settings are increasingly becoming resistant to commonly used antibiotics. Prevalence of extended spectrum β-lactamases in clinical *Klebsiella pneumoniae* has been reported to be 45-80% in Tanzania [1,2]. While *bla*_CTX-M-15_ is the most common ESBL allele among *Escherichia coli* in Tanzania [3-5], little is known about ESBL alleles in *K. pneumoniae*. In Tunisia *bla*_CTX-M-15_ was the predominant allele among *K. pneumoniae* isolates and was found in association with IncF plasmids [6]. The excessive use of cephalosporins in clinical practice has resulted in an increase of gram-negative enteric bacteria resistant to these drugs. ESBL genes responsible for resistance are mainly found in mobile genetic elements that can readily spread through bacterial populations [7-9]. ESBL –producing *K. pneumoniae* usually express resistance to various antibiotics [3,6-9], therefore their antibiotic therapy is limited to a few expensive drugs which, in most cases, are not available in developing countries.

The distribution of ESBL alleles varies among countries. The isolation of *bla*_SHV_- and *bla*_CTX-M_-producing *K. pneumoniae* has been demonstrated in different multicentre studies [10] and, as in *E. coli*, the dissemination of ESBLs is due to clonal expansion and/or plasmid transfer [11,12]. Dissemination of major *bla*_CTX-M-15_ producing *K. pneumoniae* epidemic clones ST11, ST15, ST147 and ST258 has been reported in Europe, North America and Asia [11,12]. Very few studies have reported on the problem of ESBL producing *K. pneumoniae* in Africa especially in sub-Saharan Africa [4,5,13]. In Tanzania *bla*_CTX-M-15_ has been demonstrated in one strain of *K. pneumoniae*[5]. Despite the high prevalence of these isolates in nosocomial infections, large studies to investigate the molecular epidemiology of these isolates in Tanzania are still lacking. We therefore conducted an epidemiological study to determine the molecular epidemiology of ESBL producing *K. pneumoniae* within the largest tertiary hospital in the Lake Zone of Tanzania.

## Methods

### Bacterial strains and susceptibility testing

Between April 2009 and March 2010 a total of 1260 clinical specimens were processed of which 700 were from adults, 600 from neonates and 60 from children. One hundred and three non repetitive ESBL producing *Klebsiella pneumoniae* were obtained from these routine clinical specimens; [blood (61), wound swab (13), urine (12) and pus (6)] were analyzed. All inpatient’s specimens were obtained after a hospitalization time of more than 72hrs. The specimens were processed using standard operative procedures; *K. pneumoniae* isolates were identified using in-house biochemical profiles which included: Triple-Sugar-Iron, Urease, Voges-Prosgauer, Methyl-Red, citrate utilization, indole production and motility. In case of ambiguous results a further identification using API 20E (BioMerieux, France) was done following the instructions of the vendor. Antibiotic susceptibility was determined using disk diffusion method on Mueller-Hinton agar (Oxoid, Thermo Scientific, UK) as recommended by the Clinical and Laboratory Standard Institute (CLSI) [14]. Susceptibility was tested to 11 antibiotics: ampicillin (10 μg), amoxicillin/clavunate (20/10 μg), tetracycline (30 μg), gentamicin (10 μg), sulfamethoxazole-trimethoprim (1.25/23.75 μg), ciprofloxacin (5 μg), ceftazidime (30 μg), cefepime (30 μg) and meropenem (10 μg) (OXOID, Thermo Scientific, UK). Quality control was performed using *Escherichia coli* ATCC 25922 reference strain. ESBL screening was performed using MacConkey agar with 30 μg/ml cefotaxime and then confirmed using double disk synergy (Disk approximation method) [9]. *Escherichia coli* ATCC 25922 was utilized as negative control whereas *Klebsiella pneumoniae* ATCC 700603 was used as ESBL positive control.

### Characterization of ESBLs genes

The presence of ESBL genes was determined by PCR and sequencing using primers targeting *bla*_TEM_, *bla*_SHV_ and *bla*_CTX-M_ as described previously [9]. Briefly, strains were grown on Luria- Bertani (LB) agar plates with 30 μg/ml cefotaxime and a single colony was used as template in 50 μl PCR mixture as previously described [9]. In all transconjugants the presence of tnpA/IS*Ecp*1 (1.881 kb) was tested by PCR using primers (*tnp*A F: 5’-GCAGGTGATCACAACC-3’ and *tnp*A R: 5’-GCGCATACAGCGGCACACTTCCTAAC-3’) as previosuly described [9]. All PCR products were sequenced (LGC genomics GmbH, Berlin, Germany) using the same primers plus an additional set of primers (CTF-F: 5’-GACAGACTATTCATGTTGTTG-3’ and CTF-R:5’-CGATTGCGGAAAAGCACGTC-3’) designed in this study to cover mutations that differentiate *bla*_CTX-M-15_ from *bla*_CTX-M-28_[15]; the resulting sequence was compared with known sequences using DNASTAR software (DNASTAR Inc, Madison, USA) and the NCBI BLAST algorithm.

### Pulsed-field Gel Electrophoresis (PFGE), phylogenetic group typing and MLST

Clonal relatedness between the different strains was studied using PFGE which was performed according to the Pulse Net protocol of the Centre for Disease Control and Prevention (Atlanta, USA). The agarose-embedded DNA was digested with the restriction endonuclease XbaI (Fermentas, Germany) at 37°C for 18hrs. Electrophoresis was conducted using CHEF Drive II (Bio-Rad, UK); conditions were 6V, with 5s-50s pulses for 26hrs. Band patterns were compared using Gelcompar II (Applied Maths, Belgium). Patterns were normalized using a molecular weight marker (Lambda Ladder PFGE Marker, New England Biolabs, USA) [3,9].

Phylogenetic grouping was performed using a rapid method combining *gyr*A PCR- restricted fragment length polymorphisms analysis (RFLP), *par*C-PCR and adonitol fermentation as described previously [16]. MLST was performed as described previously for 15 selected isolates based on PFGE clusters [17] (http://www.pasteur.fr/recherche/genopole/PF8/mlst/Kpneumoniae.html). Briefly, PCR for seven housekeeping genes; *rpo*B, *gap*A, *mdh*, *pgi*, *pho*E, *inf*B and *ton*B was conducted and products directly sequenced. Analysis was carried out as described on the website.

### Location of ESBL genes

Conjugation was performed to investigate the transferability of the resistance determinants using plate mating experiments as described previously [9]. Eighteen *K. pneumoniae* from different PFGE clusters were used as donor and *E. coli* K12 CC118 (Rif^R^, Str^R^, Lac-, plasmid-free) as a recipient strain. Transconjugants were selected on Luria Bertani/ lysogeny broth (LB) agar plates supplemented with 30 μg /mL cefotaxime and 300 μg /mL rifampicin and were confirmed for the presence of ESBL genes phenotypically, using the double disk synergy test (DDST) disk approximation method and genotypically, by testing for the presence of the respective ESBL gene via PCR. The frequency of conjugation was expressed relative to the number of donor cells.

Plasmid sizing was performed on clinical isolates and transconjugants using S-1 nuclease PFGE as described previously [9]. Southern blotting was performed using overnight capillary transfer (CUMC Protocol for Southern Blot, New York) and hybridization was accomplished using a *bla*_CTX-M-15_ DIG labelled probe (DIG High Prime DNA Labelling and Detection Starter Kit II, Roche, Germany) following the manufacturer’s instructions.

PCR based replicon typing with confirmatory sequencing was carried out with selected isolates and their transconjugants using primers pairs which recognize FIA, FIB, FII, FrepB, I1, P, A/C, X, HI1,HI2, L/M, FIC, Y, W,T, K and N replicons [7].

### Ethical approval

The study was approved by the Bugando Medical Center/Catholic University of Health and Allied Sciences ethics committee and drug susceptibility results were used for routine patient care.

## Results

### Bacterial strains and susceptibility pattern

**Figure 1:**
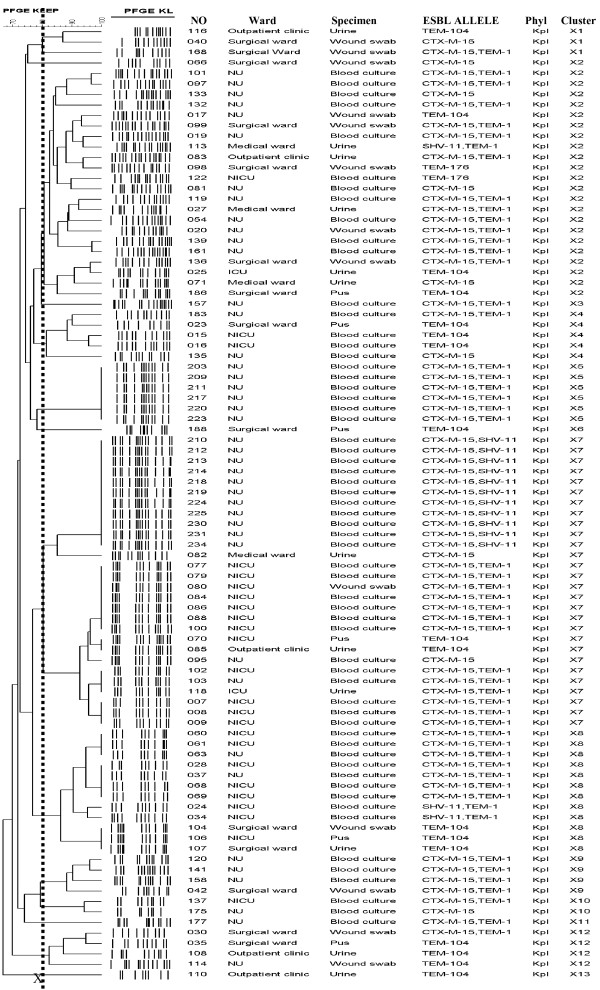
**PFGE dendrogram of ESBL–producing *Klebsiella pneumoniae*.** The PFGE patterns of the 92 *K. pneumoniae* ESBL producers are displayed on the dendrogram. The diagram also shows the isolate numbers, wards, specimens, ESBL alleles, phylogenetic groups as well as the PFGE cluster. Dashed X line indicates SAB of 0.8 revealing 13 clusters (X1-X13). NU, neonatal unit; NICU, neonatal intensive care unit.

A total of 92 *K. pneumoniae* isolates were confirmed to be ESBL producers, they represented 50.3% of all *K. pneumoniae* isolated over a period of 12 months. Most of *K. pneumoniae* producing isolates were from inpatients 87 (94%). The majority of isolates were recovered from blood culture samples from neonatal unit and neonatal ICU (NICU) 59 (64%) (Figure 1). Nineteen isolates (21%) were from wound swabs and pus and twelve (13%) were isolated from urine specimens from various wards (Figure 1). A higher rate of resistance to commonly used non-beta lactams was observed. All isolates were resistant to gentamicin and sulphamethaxazole/trimethoprim; the rate of resistance to tetracycline and ciprofloxacin was 98% and 54% respectively. A total of 25 (38%) isolates from neonatal unit and NICU were resistant to ciprofloxacin compared to 17 (68%) of isolates from other wards (chi-square 5.29, (p = 0.02)). All isolates were sensitive to meropenem using the disc diffusion test (Table 1).

**Table 1:** **Susceptibility profile of 92 ESBL producing *Klebsiella pneumoniae *isolates**

**Antibiotic**	**%resistance**	**Mean zone diameter ± SD: resistance**	**Mean diameter ± SD: sensitive**
Ampicillin (30 μg)	100%	6.06 ± 0.2356 mm	NA
Amoxicillin/Clavulanate(20/10 μg)	100%	10.7 ± 0.428 mm	NA
Tetracycline (30 μg)	98%	6 ± 0.000 mm	17 mm
Gentamicin (10 μg)	100%	6.22 ± 0.428 mm	NA
SXT(1.25/23.75 μg)	100%	6.56 ± 0.856 mm	NA
Ciprofloxacin (5 μg)	54%	8.67 ± 0.485 mm	23.44 ± 0.511 mm
Ceftazidime (30 μg)	100%	13.78 ± 0.428 mm	NA
Cefepime (30 μg)	95.7%	11.3 ± 2.6 mm	22.0 ± 2.8 mm
Meropenem (10 μg)	0.00%	NA	24.4 ± 0.3 mm

NA = not applicable.

### ESBL alleles

Of 103 phenotypically confirmed ESBL producing *Klebsiella pneumoniae* isolated during study period 92 (89%) were PCR-positive using the primers (CTX-M, TEM, SHV) described in the materials and methods section. Following PCR and sequencing *bla*_CTX-M-15_ was the commonest ESBL allele detected. It was found in 70 (76%) of cases. In a majority of isolates 49 (70%), the *bla*_CTX-M-15_ allele occurred in combination with *bla*_TEM-1_. Also *bla*_CTX-M-15_ occurred in combination with *bla*_SHV-11_ in 11 (16%) isolates. Other ESBL alleles detected were *bla*_TEM-104_ (18%) and *bla*_TEM-176_ (2%). *Bla*_CTX-M-15_ was the most frequent allele (87%) among isolates from neonatal unit and NICU (Figure 1). In all 18 strains tested the IS*Ecp*1/*tnp*A element was found upstream of *bla*_CTX-M-15_^.^

### Genetic relatedness

The PFGE-based patterns of the isolates were assigned to 13 clusters using a similarity index (SAB) of 0.8 Figure 1. These clusters contained sub-clusters, as shown in Figure 1.Cluster X5 which was assigned to ST48 was found to be clonal. The cluster X7 contained 2 large sub-clusters each with identical strains; one sub-cluster was assigned to ST48 and the other to ST14. All these sub-clusters with identical strains occurred in the neonatal unit and NICU, thus representing outbreaks in these units. The first outbreak occurred in June 2009 isolates in cluster X8, followed by sub-clusters in X7 in January and March 2010. The largest cluster was cluster X7 which contained 28 isolates of which 26 (93%) were from the neonatal unit and NICU. The isolates in this cluster have a single differing fragment thus indicating their close relatedness. The cluster X2, as the second largest, contained diverse isolates from various wards and could further be divided into 4 sub-clusters. Representative strains from this cluster were grouped to ST101 and ST147. Cluster X9 with 6 isolates was assigned to ST348. All *K. pneumoniae* isolates were grouped in the phylogenetic group KpI using *gyr*A PCR-RFLP.

### Location of *bla* CTX-M-15 genes and transferability of resistance

**Figure 2:**
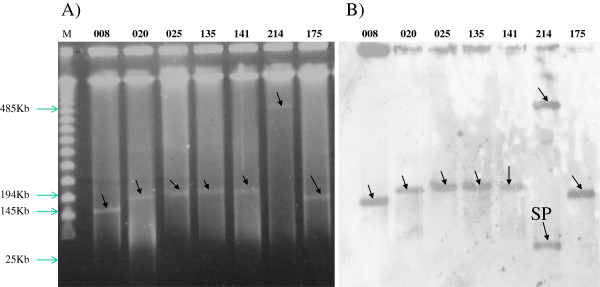
**S1 nuclease PFGE-based sizing of large plasmids for 8 clinical isolates. A)** Agarose gel showing S1 nuclease PFGE-based sizing of large plasmids for 8 isolates. M indicates the Lambda molecular weight marker. Plasmid size preparations from isolate number 08, 20, 25, 135, 141, 214 and 175 reveal plasmids with sizes ranging from 25 kb to 485 kb which are indicated with arrowheads; **B)** shows the corresponding gel after southern blotting and DIG hybridization. Hybridized plasmids are indicated with arrowheads. A small plasmid is labeled with SP; its size of 25 kb was determined after digestion with HindIII, BamHI and EcoRI (data not shown).

Sixteen of 18 *Klebsiella pneumoniae* isolates, which were randomly selected as donors, were able to transfer resistance at a frequency of 10^-3^-10^-7^ transconjugants per donor cell (Table 2). Different sizes of plasmids were found in clinical isolates ranging from 25 kb-485 kb in size and all were found to hybridize to the DIG labelled *bla*_CTX-M-15_ probes. A commonly identified 145 kb IncF plasmid was detected in 8 (44%) of the isolates tested. A representative isolate from 11 identical strains in cluster X7 contained 2 plasmids (485 kb and 25 kb). Interestingly this isolate did not transfer resistance by conjugation and both plasmids gave positive signals on hybridization (Figure 2). IncF replicons were found in 12 isolates (66.6%). Gentamicin resistance was transferable in all conjugative cases, Gentamicin-co-trimoxazole (GM-SXT) in 7 (38%), and GM-SXT-TET (Tetracycline) in 3 (16%) cases.

**Table 2:** **Phenotypic and molecular characteristics of 18 representative strains of *Klebsiella pneumoniae *used as donors**

**S NO**	**Isolate**	**ESBL allele**	**Inc group**	**PFGE cluster**	**ST**	**Phyl**	**Conjugation frequency**	**Plasmid size**	**Transferable resistance**
1	020	CTX-M-15, TEM-1	FII	X2	ST147	KpI	10-5	145 kb	GM,SXT
2	019	CTX-M-15, TEM-1	FII	X2	ND	KpI	10-7	194 kb	GM
3	025	TEM-104	FII	X2	ST101	KpI	10-7	145 kb	GM,SXT
4	024	TEM-104	ND	X8	ST14	KpI	10-6	194 kb	GM
5	028	CTX-M-15, TEM-1	FII	X8	ST14	KpI	10-6	145 kb	GM
6	175	CTX-M-15	FII	X10	ND	KpI	10-5	97 kb	GM
7	071	CTX-M-15, TEM-1	ND	X2	ST101	KpI	10-6	485 kb	GM
8	008	CTX-M-15, TEM-1	FIA	X7	ST14	KpI	10-5	97 kb	GM
9	107	TEM-104	FII	X8	ST14	KpI	10-5	194 kb	GM
10	108	TEM-104	FIA	X12	ND	KpI	10-3	97 Kb	GM
11	214	CTX-M-15, TEM-1	ND	X7	ST48	KpI	Neg	485 Kb, 25 kb	-
12	120	CTX-M-15, TEM-1	FII	X9	ST348	KpI	10-6	145 kb	GM,SXT
13	133	CTX-M-15	FII	X2	ND	KpI	10-7	145 kb	GM,SXT,TET
14	135	CTX-M-15	FII	X4	ND	KpI	10-3	145 kb	GM,SXT, TET
15	141	CTX-M-15, TEM-1	ND	X9	ST348	KpI	10-5	145 kb	GM,SXT
16	211	CTX-M-15,TEM-1	ND	X5	ST48	KpI	Neg	-	-
17	118	CTX-M-15	ND	X7	ST14	KpI	10-7	97 kb	GM,SXT
18	081	CTX-M-15	FII	X2	ND	KpI	10-6	145 kb	GM

Inc = Incompatibility; ST = Sequence Type; Phyl = Phylogenetic group; S NO = Strain number; GM = gentamicin; SXT = sulfamethoxazole-trimethoprim; TET = tetracycline.

ND = not determinable.

## Discussion

The results of this study provide insights into the molecular epidemiology of ESBL producing *K. pneumoniae* isolates in a single tertiary hospital in Tanzania, thereby representing the first large-scale study to characterize ESBL producing *K. pneumoniae* in Tanzania. A high rate of ESBL producing *K. pneumoniae* isolates is observed as described in various studies [18-21]. The majority of the isolates characterized in this study were from the neonatal- and its intensive care- units. Infections caused by ESBL-producing organisms in neonates are usually reported to be hospital-acquired and associated with invasive procedures [20,22,23]. The predominance of these isolates in these units could be explained by the high use of the third generation cephalosporins, as cefotaxime was the most prescribed drug in the unit during study period. This could further be supported by a significantly (p < 0.05) low rate of resistance towards ciprofloxacin in the neonatal unit and NICU compared to the other wards as ciprofloxacin is usually contraindicated in neonates and children. These units have a high rate of empirical treatment using ampicillin plus gentamicin as first line and cefotaxime as second line antibiotic treatments [20]. As in other studies [22,24] all isolates were found to be resistant to gentamicin and in all cases gentamicin resistance was transferable by conjugation, thus eliminating gentamicin as a treatment option for ESBL producing *K. pneumoniae*. All isolates were sensitive to meropenem but due to high costs this drug is not commonly used in this hospital.

There is a significant difference in the isolation rate of ESBL-producing *K. pneumoniae* between this study and other studies done previously in Tanzania. This may be due to a variation in the epidemiology of ESBL producing organisms between the hospitals [10]. As in previous studies [25-27] the *bla*_CTX-M-15_ allele associated with ISEcp1 was carried on multiple conjugative plasmids of different sizes ranging from 25 kb-485 kb, with one clone having multiple copies of *bla*_CTX-M-15_ genes, one copy in the chromosome and two other copies in a 485 kb and a 25 kb plasmid This reflects transmission dynamics of this element, comprising of intra strain mobility on different genetic entities (chromosome, plasmids) within a single isolate, in addition to inter- strain and inter-species transmissibility. Multiple ESBL alleles were observed in which *bla*_CTX-M-15_ and *bla*_SHV-11_ co-existed in one clone in the neonatal unit; this has recently been described in clinical isolates in Poland as a new strategy of *K. pneumoniae* to confer resistance to antibiotics [28]. There is an antibiotic selection advantage for the isolates with multiple alleles and multiple copies of genes for an allele as seen in cluster X7 in this study.

A high diversity of ESBL producing *K. pneumoniae* was observed using PFGE and isolates could be divided in 13 clusters at 80% similarity with multiple sub-clusters. In contrast MLST of three large clusters with 46 isolates mainly involved in neonatal sepsis revealed three sequence types 14, 48 and 348; compared to previous reported ST11 and ST15 [19,27,29]. The detection of *K. pneumoniae* ST14 and ST48 carrying *bla*_CTX-M-15_ in a single hospital associated with neonatal nosocomial infection are the first description of the sequence types on the African continent. ST14 and ST48 carrying *bla*_CTX-M-15_ could be the predominant clones in Africa, a finding which has to be clarified by further studies from different African countries and underscores the need for joint efforts to survey ESBL-producing isolates in Africa. Recently in Oman a ST14 isolate has been found to harbor *bla*_CTX-M-15_ and *bla*NDM-1, also a ST14 *K. pneumoniae* carrying *bla*_CTX-M-15_ with a similar resistance pattern as described here has been isolated in two patients in Spain from rectal swabs [27]. ST147 and ST101 were detected in cluster X 2 which is the most diverse cluster. These sequence-types have been reported to occur worldwide and are associated with multi-resistant *K. pneumoniae*[28,29].

Clonal outbreaks caused by ST14 and ST48 were discernible by PFGE. All outbreaks occurred in the neonatal unit and neonatal ICU at different time-points and could be due patient to patient transmission or the acquisition from a common source (contaminated equipments) or from healthcare workers [28-30]. Different clones were involved in all three outbreaks; this further supports the high diversity of these strains in this setting, and represents a huge challenge for the local hospital infection control team. Constant surveillance, sustainable hygiene measures and accurate detection in the hospital are crucial for a quick and appropriate management of these outbreaks [31]. This approach has been used in most developed countries, but can be difficult to implement in a country like Tanzania where availability of even routine culture and susceptibility testing is a major challenge in many hospitals. There is also a need for the hospitals to institute an antibiotic policy guided by antimicrobial stewardship measures such as adaption of empirical treatment regimens to hospital epidemiological susceptibility data. Despite of the fact that these isolates were collected 2009–2010, no major intervention could be instituted until now to control the problem and therefore molecular epidemiology of these isolates is expected to have remained more or less unchanged for the past 3 years.

## Conclusion

This study highlights the need to establish an antimicrobial resistance surveillance network in Tanzania for *K. pneumoniae* and other ESBL-producing *Enterobacteriaceae* to monitor the trends and new types of resistance mechanisms emerging in hospitals. Also, the factors responsible for the selection and dissemination of this plasmid encoding the *bla*_CTX-M-15_ and multiple clones carrying *bla*_CTX-M-15_ should be considered for the current clinical management and antibiotic policies, investigated further and controlled to prevent major outbreaks in future.

## Competing interests

The authors declare that they have no competing interests.

## Authors' contributions

SEM, EFL, CI, ED and TC designed the study, SEM and CI performed the experiments, SEM, CI, TC analyzed the data, TH analyzed sequences, SEM, CI, ED and TC wrote the manuscript which was corrected and approved by all the other coauthors. All authors read and approved the final manuscript.

## Pre-publication history

The pre-publication history for this paper can be accessed here:

http://www.biomedcentral.com/1471-2334/13/466/prepub
